# Functional Foods and Adapted Physical Activity as New Adjuvant Therapy for Chronic Kidney Disease Patients

**DOI:** 10.3390/nu16142325

**Published:** 2024-07-19

**Authors:** Giulia Marrone, Arianna Murri, Silvia Urciuoli, Manuela Di Lauro, Elisa Grazioli, Pamela Vignolini, Kevin Cornali, Eliana Tranchita, Claudia Masci, Claudia Cerulli, Luca Di Marco, Anna Paola Mitterhofer, Attilio Parisi, Annalisa Noce

**Affiliations:** 1 Department of Systems Medicine, University of Rome Tor Vergata, 00133 Rome, Italy; cornali.kevin@hotmail.it (K.C.); masciclaudia@gmail.com (C.M.); luca.dimarco_1969@libero.it (L.D.M.); annapaola.mitter@uniroma2.it (A.P.M.); annalisa.noce@uniroma2.it (A.N.); 2 Department of Exercise, Human and Health Sciences, Foro Italico University of Rome, 00135 Rome, Italy; 3 Department of Statistics, Computer Science and Application—PHYTOLAB (Pharmaceutical, Cosmetic, Food Supplement, Technology and Analysis), University of Florence, Sesto Fiorentino, 50019 Florence, Italy; silvia.urciuoli@unifi.it (S.U.); pamela.vignolini@unifi.it (P.V.); 4 UOSD Nephrology and Dialysis, Policlinico Tor Vergata, 00133, Rome, Italy

**Keywords:** functional foods, sarcopenia, circular economy, non-communicable diseases, extra virgin olive oil, natural bioactive compounds

## Abstract

Background: Chronic kidney disease (CKD) will become the fifth leading cause of death in the world by 2040. It is fundamental to prevent and treat this pathology to reduce its impact on national health costs. This trial’s aim is to evaluate the effects induced by a combination of consumed functional foods (FFs) with adapted physical activity (APA) on the progression of CKD-related comorbidities. Methods: The study lasted 12 weeks. We divided 40 CKD patients into four groups: mixed (FF + APA), APA, FF and control group (usual care). The FFs were characterized by their total antioxidant capacity and antiradical activity. The APA was performed though an online training protocol, three times per week, 1 h each session. Results: At the end of the study, we observed, in the mixed group, a decrease in azotemia (*p* = 0.0272), diastolic blood pressure (*p* = 0.0169), and C-reactive protein (*p* = 0.0313), with increases in the FORD test (*p* = 0.0203) and fat free mass (*p* = 0.0258). The APA group showed a reduction in total cholesterol (*p* = 0.0039). Conclusions: The combination of FFs and APA can help counteract several CKD-related comorbidities, such as arterial hypertension, dyslipidemia and uremic sarcopenia, and improve the CKD patients’ quality of life.

## 1. Introduction

Chronic kidney disease (CKD) constitutes one of the most important causes of death in the 21st century, due to the increasing prevalence of its risk factors and its comorbidities that significantly impact the quality of life (QoL) of nephropathic patients [1]. It is estimated that approximately 843.6 million individuals worldwide were affected in 2017 [2]. In addition to arterial hypertension, type II diabetes mellitus and cardiovascular diseases, uremic sarcopenia (US) also plays a pivotal role in the worsening of CKD patients’ clinical conditions. The mechanisms involved in US are several and include activation of the ATP-dependent ubiquitin-proteasome system, physical inactivity and comorbidities typical of uremia, namely metabolic acidosis, low-grade chronic inflammatory state, vitamin D deficiency, insulin resistance, hormonal impairments and gut dysbiosis [3]. It is certain that all of these mechanisms converge towards a final process of increased protein degradation and reduced protein synthesis, more aggressively than that which occurs in the physiological aging process [4,5]. As reported by the updated guidelines of the European Working Group on Sarcopenia in Older People (2019), sarcopenia is defined as a decrease in muscle strength and muscle mass, associated or not with a worsening in physical performance, a parameter used to stage the severity of this pathology [6,7]. In CKD patients, sarcopenia is often associated with mineral bone disorders [8].

US and CKD mineral bone disorders are associated with a worsening of CKD patients’ QoL [9] and with an increased risk of mortality and morbidity for all causes [7,10,11], resulting in the higher number of hospitalizations and in a longer length-of-stay [3]. All these factors are associated with depression and demotivation of CKD patients to incorporate physical exercise into their daily lives, leading to an exacerbation of the muscle protein catabolism and loss of strength, physical performance and aerobic capacity (VO2 peak) [12].

Over the years, new therapeutic strategies based on the consumption of functional foods rich in natural bioactive compounds (NBCs), such as polyphenols, and on adherence to adapted physical activity (APA) protocols have been discussed both to improve CKD patient QoL and to reduce the costs burdening the national health system for their clinical management [13]. 

In this regard, many studies demonstrate the direct association between the intake of foods rich in NBCs and human health. In fact, the class of polyphenols, which are secondary metabolites present in all plants, plays a protective role for the plants and for humans [14,15,16]. Over the last 20 years, the scientific community has studied the effects of polyphenols on human health, showing their potential use in the prevention and treatment of many chronic degenerative non-communicable diseases and their related complications. There is evidence of risk reduction and prevention of cardiovascular diseases, some types of cancer, diabetes mellitus, CKD, osteoporosis, gastrointestinal diseases, chronic obstructive pulmonary disease and neurodegenerative diseases [17,18,19,20,21,22,23,24,25]. NBCs can also exert chemo-preventive effects by eliminating carcinogens, modulating tumor cell signaling pathways and cell cycle progression and promoting apoptosis [26,27] 

A 2023 review explains how polyphenols reduce muscle soreness and accelerate the recovery of muscle function after exercise. However, there is no relevant evidence on the effect of polyphenols on physical performance and body composition of sarcopenic patients [28]. In this context, two functional bars based on fruit, vegetables, extra virgin olive oil (EVOO) and innovative ingredients (micronized grape pomace, micronized grape seeds, olive leaf powder) derived from the recovery of waste from agri-food supply chains were selected for this study. These ingredients were selected to evaluate the possible positive effects of foods rich in polyphenols in CKD patients. EVOO was the first ingredient to be chosen for its well-known beneficial effects on human health, thanks to its NBC content, including hydroxytyrosol, tyrosol, oleuropein aglycone, oleacin and oleocanthal. Previous studies by our research group have allowed us to demonstrate the positive effect of an EVOO rich in NBCs in the management of CKD patients [18,29,30,31,32]. For this reason, in addition to EVOO, we have chosen innovative ingredients, rich in bioactive molecules, which are derived from the circular recovery of waste and by-products from the EVOO and wine production chain. 

Many studies highlight the beneficial effects of *Olea europea* L. on human health, including antioxidants and antimicrobial activity, the positive modulation of gut microbiota, neuroprotective effects, adjuvant to anti-tumor therapy, cardioprotective action and the modulation of glucose metabolism [18,29,30,32,33]. Other innovative ingredients, selected for functional food bars, were grape pomace and seed, which represent the most abundant waste from winemaking and an important source of bioactive molecules that show antioxidant, anti-inflammatory, cardioprotective and hepatoprotective effects and hypoglycemic and neuroprotective actions [32,34]. These findings suggest that secondary raw materials, obtained by a circular economy model, could represent innovative and functional ingredients for new food production. These ingredients exert positive effects on human health and have a sustainable impact on the environment. 

In addition to NBCs, an increasing number of studies are highlighting the importance of APA protocols in CKD patients. Physical exercise should be performed as an anabolic energy supply intervention [35]. Resistance training (RT), targeted for increased muscle strength and hypertrophy, seems to be effective and protective during a low protein diet to avoid the worsening of protein-energy wasting while aerobic training (AT) seems to ameliorate the VO2 peak, the microvascular circle and endothelial function [36,37]. The combination of both training regimens seems to reduce muscle weakness through significant improvements in muscular strength and in quadriceps muscle volume, which is particularly important for preventing sarcopenia in CKD patients [38]. Despite this knowledge, adherence to nutritional therapy and pharmacological treatment is low in CKD patients, ranging from 20% to 70%, and sedentary behavior is widespread among this population [39,40]. According to recommendations [41,42], new strategies are necessary to increase adherence to a positive lifestyle in CKD patients. Pathways, individualized and adapted to patients through a multidisciplinary approach, seem to be necessary to manage CKD [39]. Moreover, according to our knowledge, there are no studies about the effect of a tailored APA protocol combined with functional foods (FFs) on CKD patients.

Thus, the purpose of our study is to assess the possible beneficial effects, induced by the combination of FFs consumption, characterized by high anti-inflammatory and antioxidant power, with an APA protocol, on the CKD patients QoL and on the progression of CKD- related comorbidities.

## 2. Results

In Table 1, the anthropometric and epidemiological features of the study populations at T0 are reported.

The following five tables illustrate the results of our clinical trial. Each table shows the different examined parameters, analyzed during all time-points of the study. The study group was divided into four subgroups (namely mixed, APA, FFs and control group), and all parameters were collected at T0 (baseline) and T1 (after a 12-week study). The laboratory parameter findings are reported in Table 2. Regarding the renal function biomarkers, at the end of the study, in the mixed group, we observed a statistically significant reduction in azotemia (59.11 ± 15.02 mg/dL vs. 54.00 ± 14.15 mg/dL, *p* = 0.0272). Regarding the lipid profile, in the APA group, we highlighted both a statistically significant reduction in total cholesterol (TC) levels and a statistically significant increase in high-density lipoprotein (HDL) cholesterol levels, respectively, of 239 (85–333) mg/dL vs. 200 (69–282) mg/dL (*p* = 0.0039) and 56.5 (37–75) mg/dL vs. 50.5 (31–73) mg/dL (*p* = 0.0156). 

The arterial BP parameter findings are reported in Table 3. At the end of the study, only in the mixed group, we pointed out a statistically significant reduction in diastolic BP values (88.50 ± 15.28 mmHg vs. 78.00 ± 9.77 mmHg, *p* = 0.0169). Systolic BP did not show any relevant difference. 

The biomarkers of inflammation and oxidative stress are reported in Table 4. At T1 (after 12 weeks), we observed only in the mixed group both a statistically significant increase in the total antioxidant capacity (TAC) of the plasma, detected by free oxygen radicals defense (FORD) test (1.41 ± 0.47 mmol/L Trolox equivalents vs. 1.89 ± 0.54 mmol/L Trolox equivalents, *p* = 0.0203), and a statistically significant reduction in C- reactive protein (CRP) (3.8 (0.4–9.5) mg/L vs. 1.9 (0.4–5 mg/L), *p* = 0.0313).

The anthropometric parameters, the body composition evaluation and the ultrasonographic examination are reported in Table 5. At the end study, we did not observe a statistically significant modification in anthropometric parameters in any group of the study. 

Regarding body composition parameters detected by bioelectrical impedance analysis (BIA), at the end of the 12-week trial, in the mixed group we observed a statistically significant increase both in reactance (Xc) and in fat free mass (FFM) values, respectively 39.80 ± 5.67 Ω vs. 45.40 ± 8.03 Ω (*p* = 0.0232) and 69.36 ± 7.27 Ω vs. 74.14 ± 5.55 Ω (*p* = 0.0258). In the APA group, however, we highlighted both a statistically significant reduction in resistance (Rz) value and a statistically significant increase in body cell mass (BCM) value, respectively 518.78 ± 74.71 Ω vs. 479.00 ± 47.22 Ω (*p* = 0.0491) and 51.34 ± 1.91% vs. 54.27 ± 3.69% (*p* = 0.0270). 

Finally, regarding the ultrasonography examination, at T1, we pointed out in the APA group a statistically significant increase on both left and right quadriceps rectus femoris thickness (QRFT) at 1/2, respectively 1.58 ± 0.15 cm vs. 1.77 ± 0.25 cm (*p* = 0.0465) and 1.46 ± 0.32 cm vs. 1.76 ± 0.48 cm (*p* = 0.0255), and on the left QRFT at 2/3, 1.59 ± 0.30 cm vs. 1.79 ± 0.34 cm (*p* = 0.0480). On the contrary, we highlighted in the control group a statistically significant reduction on the right QRFT at 2/3, 1.57 ± 0.44 cm vs. 1.41 ± 0.43 cm (*p* = 0.0435).

The functional tests used to assess patients’ muscle strength and physical performance are reported in Table 6. 

Regarding muscle strength, at the end of the study, we observed a significant increase in the hand grip strength test (ST) in the mixed and FFs groups, respectively. The mixed group showed an increase in the left hand (38.83 ± 13.68 kg vs. 41.25 ± 13.79 kg, *p* = 0.0491), and the FFs group in the right hand (31.98 ± 13.99 kg vs. 34.47 ± 11.96 kg, *p* = 0.050). 

Concerning patient physical performance in the mixed group and in the APA group, at the end of the 12-week trial, we pointed out a statistically significant increase in the walking distance, evaluated through the six-minute walking test (SMWT), respectively 572.00 ± 75.98 m vs. 624.50 ± 89.15 m (*p* = 0.0002) and 603.75 ± 107.03 m vs. 636.87 ± 97.13 m (*p* = 0.0070). In addition, in the FFs group, we observed a statistically significant increase both in the short physical performance battery (SPPB) score (9.89 ± 1.83 points vs. 11.33 ± 0.87 points, *p* = 0.0316) and in the lower limb power assessment, evaluated by the stair climb power test (SCPT) (192.70 ± 97.57 W vs. 227.53 ± 109.02 W, *p* = 0.0280).

The prevención con dieta Mediterránea (PREDIMED) questionnaire, administered to patients at T0 and T1, did not show any statistically significant difference between the two study periods. Therefore, we can exclude possible bias due to the changes in the patients’ eating habits during the clinical trial. As for the health-related QoL, at the end of the study, the SF-36 questionnaire showed a statistically significant improvement in the sphere “change in health status” (*p* = 0.0328) in the APA group.

## 3. Discussion

CKD poses a significant economic burden worldwide, which increases remarkably with the disease progression towards end-stage renal disease where renal replacement therapy is mandatory. Suffice it to say that the annual direct costs associated with the clinical management of CKD patients increase approximately 20–25 times for patients on renal replacement therapy, compared to patients on conservative therapy [43]. In order to reduce the global burden of CKD, innovative methods involving a multidisciplinary approach, based on close collaboration between medical and non-medical figures, is of fundamental importance. In this respect, the same KDIGO guidelines updated to 2024 emphasize that CKD care models must be based on a multidisciplinary care team [42]. However, in this original study and in our previous publications, we showed how APA experts, such as clinical kinesiologists, should also take part in the multidisciplinary team for the clinical management of nephropathic patients, in order to improve their QoL. 

Moreover, in the field of innovative approaches, adherence to the UN Agenda 2030 for Sustainable Development is of considerable importance. In particular, we refer to goal 3, namely “Ensure healthy lives and promote well-being for all at all ages”, whose 3.4 target thus reads: “By 2030, reduce by one third premature mortality from non-communicable diseases through prevention and treatment and promote mental health and well-being” (Transforming our world: the 2030 Agenda for Sustainable Development) [44]. However, in our in vivo study, we decided to consider another Agenda 2030 Sustainable Development Goal (SDG). In fact, by prototyping and formulating FFs, enriched with the co-products of the wine and oil chain, obtained through a circular economy model, we were able to comply with SDG 12, namely “Ensure sustainable consumption and production patterns”. More specifically, we refer to the 12.5 target that reads as follows: “By 2030, substantially reduce waste generation through prevention, reduction, recycling and reuse” [44]. The FFs, administered to enrolled CKD patients, were enriched with olive leaves and micronized grape film and grape seeds, all waste products with a high content of NBCs. Numerous studies have shown how only a small portion of polyphenols is present in EVOO (<0.5%), while the remaining part is present in waste products. In fact, Cecchi et al. showed that the total amount of polyphenols present in olive leaf powder varies between 7.87 and 34.21 mg/g, while that of EVOO ranges between 2.79 and 21.03 mg/g. Mapanga et al., in a study conducted on streptozotocin-induced diabetic rats, highlighted how oleanolic acid (60 mg/kg, *per os*), an important NBC present in olive leaves, was able to decrease serum creatinine concentration and enhance eGFR [45]. 

Regarding renal function, serum creatinine showed a slight enhancement in the mixed and APA groups. These data can be partially explained by the increase in muscle mass due to physical activity. While the eGFR decreased slightly in the APA and FFs groups, it did not determine a change in the CKD stage.

Regarding other renal function parameters, we observed a decrease in azotemia in the mixed group. This result is probably due to the higher calorie intake provided by the FFs, combined with APA. In fact, as already known, in nephropathic patients, high-calorie diets, characterized by protein-controlled intake, are related to a decrease in azotemia thanks to the amelioration of protein metabolism [46]. In this context, in the FFs group, we observed a decreasing trend in azotemia levels, but this approach turned out not to be sufficient to induce an amelioration of this parameter during the observation period. Interestingly, in the mixed group, the combined approach demonstrated that the FFs, associated with physical activity, are useful in promoting protein synthesis and consequently decreasing azotemia. 

Regarding the lipid profile, we observed in the APA group a decrease in TC, while the HDL cholesterol increased. However, we did not observe this enhancement in the mixed group. It is well known that low-to-moderate physical activity is able to induce a reduction in TC [47], and to explain the improvement in TC, we have to consider that the mean value in the APA group, at baseline, was higher than that of the other three groups. Therefore, we can speculate that the significant result is attributable to the difference at T0 between the four groups. 

Regarding the inflammatory parameters, we observed a CRP reduction in the mixed group. This result should be ascribable to the caloric surplus supplied by the FFs (122 Kcal per bar), that permitted the patients to reach the higher energy requirements, taking into account the increase in energy expenditure, due to the APA. In fact, the basal metabolic requirements of CKD patients are increased [48] and, if not adequately balanced, they can induce a vicious circle, enhancing the systemic inflammation [49]. Moreover, physical activity is also a useful approach to counteract the inflammatory status [50]. As highlighted in a previous meta-analysis conducted in the general population, CRP levels dropped after a training program administration, regardless of the individuals’ sex or age [51]. These data were also stressed in the mixed group of our study.

Several studies pointed out how APA plays a pivotal role in reducing diastolic BP values. However, in the APA group, APA alone was not capable of inducing a significant reduction in this parameter. In contrast, NBCs present in waste powders and micronized products, obtained from the wine and olive oil supply chain, can potentially have an anti-hypertensive effect and, therefore, were likely to exert a synergistic effect with APA.

Furthermore, in the mixed group, the combined approach led to a significant increase in the antioxidant capacity of the organism, detected through the FORD test.

The results of this study, relating to the intake of the two bars, allow us to hypothesize a functional effect of NBCs associated with APA on human health. In particular, the positive effects may be related to the presence of EVOO rich in bioactive phenolic compounds, which constituted the lipidic fraction of the tested bars. Recent studies highlight a direct correlation between the use of EVOO and a high adherence to the Mediterranean diet (MD) with muscle function, related to the maintenance of skeletal muscle homeostasis during aging. The positive effects would be linked to the phenolic compounds present in EVOO, which could activate anabolic pathways and counteract age-related changes and diseases involved in muscle degeneration, such as mitochondrial alterations and inflammatory processes [52]. Moreover, polyphenols block proinflammatory transcription factors by interacting with proteins responsible for gene expression of the inflammation [17]. EVOO, associated with APA, plays an important role in the anti-inflammatory activity in CKD patients [53,54,55]. In our study, this observation was confirmed by the decreased CRP levels in the mixed group. As already described in a previous study by our research group, the anti-inflammatory activity could be linked to the secoiridoids of *Olea europaea* L., such as oleocanthal, oleacin and oleuropein present in EVOO and olive leaves. These molecules can inhibit the enzymes cyclooxygenase (COX)-1 and COX-2 in a dose-dependent manner [29]. In particular, as described by Beauchamp et al., oleocanthal has an action similar to that of ibuprofen, but this secoiridoid, at the same concentration of ibuprofen, inhibits the COX-1 and COX-2 enzymes more effectively [56,57].

In this study, the NBCs of EVOO and other plant ingredients allowed the phytocomplex to act synergistically with APA. In fact, the FFs have a good TAC, linked to the NBC content of EVOO, fruit, and vegetables and to innovative sustainable ingredients, obtained from circular agriculture. These FFs can be proposed as an innovative food for human health and can also be aimed at a consumer sensitive to environmental sustainability. The use of secondary raw materials, derived from the recovery of waste and by-products from the agri-food supply chains, gives a sustainable value to the bars and suggests the choice of innovative functional ingredients, recovered from other agri-food supply chains, to improve human and environmental health.

Poor physical performance and frailty are associated with an elevated death risk and disability in CKD patients; therefore, a well-tailored lifestyle intervention is widely suggested to manage CKD patients [58]. The combination of APA with a nutritional treatment appears to be a useful approach to CKD comorbidity management, mainly for US.

According to the results of this study, 12 weeks of FF consumption, combined with an online training program, seemed to improve the health-related QoL in CKD patients. In particular, both interventions can counteract the onset and the progression of US. Lower muscle strength is considered the main determinant of sarcopenia, and it is a strong predictor of adverse outcomes, such as falling and hospitalization [59]. According to our data, bar supplementation combined with physical exercise significantly improved the strength values for hand grip ST; additionally, a positive trend was detected in the group that followed only physical exercise. These results are in line with those reported in the literature, where the combination of exercise and nutritional therapy seems to be more effective than exercise alone on sarcopenia management [60]. Following only a nutritional therapy regimen could limit the physical parameters in the long term, although the FFs group showed an increase in the hand grip ST value [60]. Based on our results, achieving positive effects on muscle hypertrophy and body composition seemed to be mandatory to perform physical exercise. After 12 weeks of APA, the APA group showed a significant improvement in the QRFT and BCM%. In addition, when the exercise was combined with FF supplementation, it seemed to ameliorate the FFM% of patients as well. These results support those found in our previous pilot study on CKD patients [13]. The worsening of the QRFT, observed in the control group only, underlines the necessity of these interventions in order to counteract the decline in muscle mass. Another important aspect related to the physio-pathological condition of nephropathic patients is the progressive decline in physical capacity that can compromise basic daily activities [61]. According to the results for the FFs group, nutritional therapy alone can be useful to increase physical capacity, as observed by the SPPB and SCPT tests. However, as pointed out by the SMWT results in the mixed and APA groups, to achieve a significant improvement in aerobic capacity and endurance, it seems to be necessary to administer both functional foods and physical exercise. Therefore, in line with Kanabay et al. and Grazioli et al., a well-tailored combined supervised training regimen with aerobic and resistance exercises can help to counteract sarcopenia and several functional disabilities in CKD patients [62]. Lastly, no adverse events or drop-outs occurred in this study, underlining the effectiveness and safety of the online exercise method in the CKD population. The online modality can overcome the logistic barriers to performing regular physical activity and increase patient motivation and compliance with the exercise protocol, breaking the vicious circle induced by sedentariness [63].

The results of this study, relating to the FF intake, allow us to hypothesize a healthy effect of NBCs, associated with APA.

## 4. Materials and Methods

### 4.1. Patients

**Figure 1:**
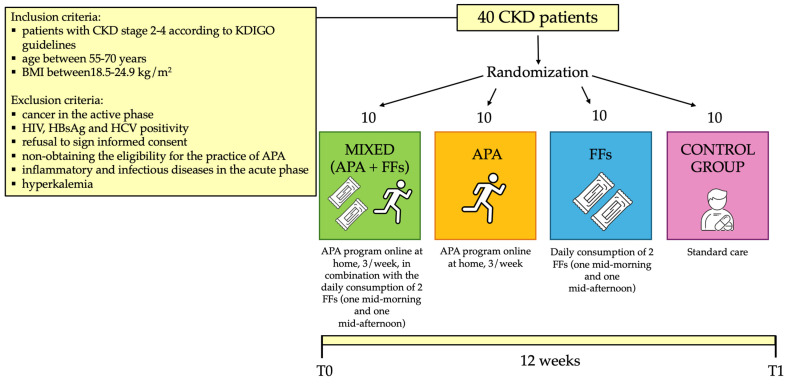
Flow-chart of the study. Abbreviations: APA, adapted physical activity; CKD, chronic kidney disease; FFs, functional foods; HBsAg, hepatitis B surface antigen; HCV, hepatitis C virus; HIV, human immunodeficiency virus.

At the Nephrology and Dialysis Unit of the University Hospital of Rome Tor Vergata, 40 CKD patients under conservative therapy were enrolled (stage 2–4, according to the kidney disease: improving global outcomes- KDIGO guidelines [42]), including 27 males (mean age 61.6 ± 8.0 years) and 13 females (mean age 63.9 ± 6.3 years). Inclusion and exclusion criteria are reported in Figure 1. 

**Figure 2:**
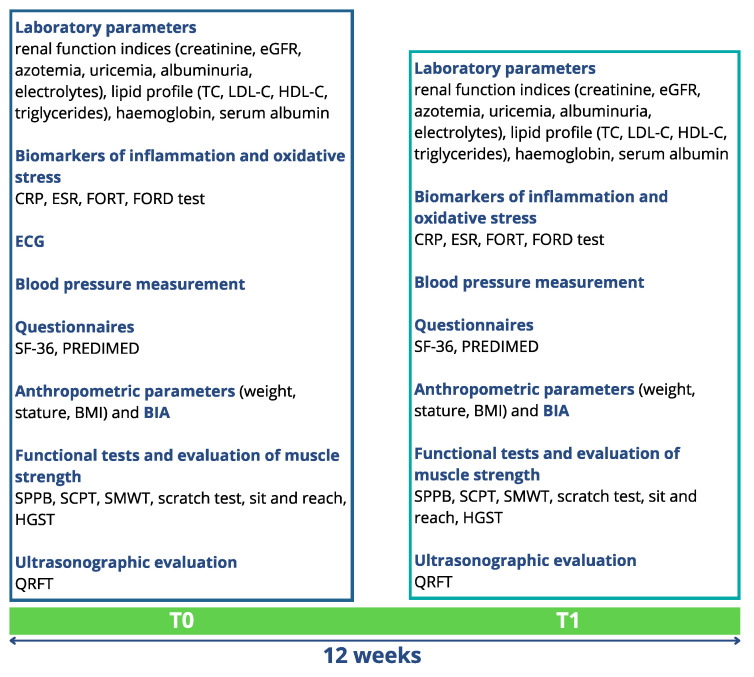
Evaluations performed at the time of enrollment (T0) and after 12 weeks (T1). Albuminuria was calculated by albumin-creatinine ratio (ACR) in a morning urine sample (spot). Abbreviations: BIA, bioimpedance analysis; CRP, C-reactive protein; eGFR, estimated glomerular filtration rate; ESR, erythrocyte sedimentation rate; FORD, free oxygen radical defense; FORT, free oxygen radicals test; HDL-C, high-density lipoprotein cholesterol; HGST, handgrip strength test; LDL-C, low-density lipoprotein cholesterol; PREDIMED, PREvención con DIeta MEDiterránea; QRFT, quadriceps rectus femoris thickness; SCPT, stair climb power test; SF-36, short form health survey-36; SMWT, six-minute walking test; SPPB, short physical performance battery; TC, total cholesterol.

Before the beginning of the study, all patients enrolled for the clinical trial signed an informed consent. The experimental protocol complied with the 1975 guidelines of the Declaration of Helsinki and was approved by the Internal Ethical Committee of Policlinico Tor Vergata. The protocol code was 223/20 of 7 December 2020. The study protocol lasted 12 weeks. The study population was divided into 4 subgroups (mixed, APA, FFs and control group), homogeneous in terms of age, gender and BMI, with 10 patients per subgroup, as described in Figure 1. The FFs group consumed two FFs (one mid-morning and one mid-afternoon) daily, and the control group represented the usual care. At the time of enrollment (T0) and at the end of the study (T1), patients underwent the evaluations reported in Figure 2. In particular, after the enrollment, all patients underwent a detailed medical examination, including anamnesis, physical examination and electrocardiogram at rest, to confirm their eligibility for the physical exercise.

### 4.2. Laboratory Parameters and Biomarkers of Inflammation and Oxidative Stress

Laboratory parameters and biomarkers of inflammation and oxidative stress were monitored at both times of the clinical trial. In particular, we evaluated indices of renal function, such as creatinine, eGFR, azotemia, uricemia, albuminuria (calculated by albumin-creatinine ratio (ACR) in a morning urine sample) and electrolytes (such as potassium, phosphorus, sodium and calcium), lipid profile (such as TC, low-density lipoprotein (LDL) cholesterol, HDL cholesterol and triglycerides), hemoglobin, albuminemia and biomarkers of inflammation (such as CRP and erythrocyte sedimentation rate (ESR)). All laboratory parameters were analyzed by Dimension Vista 1500 (Siemens Healthcare Diagnostics, Milano, Italy), except for the lipid profile, which was determined by standard enzymatic colorimetric techniques (Roche Modular P800, Roche Diagnostics, Indianapolis, IN, USA). All parameters were analyzed according to standard procedures in the clinical chemistry laboratories of the University Hospital of Rome Tor Vergata. 

For the oxidative stress evaluation and antioxidant defense assessment, all patients provided capillary blood samples, analyzed with the CR4000 photometer, using the free oxygen radicals test (FORT) and the FORD test. These colorimetric tests, through various solution-catalyzed reactions, are able to determine the concentration of reactive oxygen species and antioxidant compounds. One FORT unit corresponds to 0.26 mg/L hydrogen peroxide (H_2_O_2_). At the end of the execution of the FORT, test values below 300 U were considered “optimal” and between 300 and 330 U “borderline”, while values above 330 U indicated the presence of oxidative stress. Regarding the FORD test, values above 1.53 mmol/L Trolox equivalents were considered “optimal”, between 1.53 and 1.07 mmol/L Trolox equivalents “borderline”, and below 1.07 mmol/L Trolox equivalents “low” [64].

### 4.3. Questionnaires

At the two study time points, T0 and T1 (after 12 weeks), two questionnaires, PREDIMED and the 36-Item Short-Form Health Survey (SF-36), were administered to all patients to assess, respectively, adherence to the MD and each patient’s QoL. In our clinical trial, the PREDIMED questionnaire, designed to investigate the individual’s adherence to the MD, served to exclude possible biases due to changes in the patient’s lifestyle. This questionnaire comprises 14 items, each of which corresponds to 1 point, up to a maximum of 14 points. According to the scoring, patients were divided into 3 MD adherence groups: minimal adherence (≤5 points), medium adherence (between 6 and 9 points) and maximum adherence (≥10 points) [65]. The SF-36 is a self-administered questionnaire that assesses the patient’s QoL through the measurement of eight scales: physical functioning, role physical, bodily pain, general health, vitality, social functioning, role emotional, and mental health [66].

### 4.4. Measurement of Anthropometric Parameters and Body Composition Assessment

A Seca scale (model 700, Hamburg, Germany) with a built-in stadiometer was used to measure the anthropometric parameters of the enrolled patients, i.e., body weight (kg) and stature (m). The measurements were made to the nearest 0.01 kg for body weight and 0.1 cm for stature. The patients’ BMI was calculated as body weight divided by stature squared (kg/m^2^). For the assessment of body composition, all nephropathic patients underwent BIA using EGF Plus^®^, software Bodygram HBO (Estor, Pero, MI, Italy), based on the bioelectrical impedance vector analysis (BIVA) technology, at 50 kHz frequency. To perform the BIA, all patients assumed a supine position on a non-conductive surface, and they removed both shoes and socks to apply, on dry non-oily skin, two pairs of electrodes on the right hemisoma, one between the bony prominences of the wrist and the other between the bony prominences of the ankle. The two pairs of electrodes were applied at a 5 cm distance per pair, to which the cables were connected, the red ones to the electrodes in the distal position and the black ones to the electrodes in the proximal position. Finally, the patient placed their legs at an angle of approximately 45° and their arms at about 30° from the body. For the BIA evaluation, we considered the following parameters: resistance (Rz, Ω), reactance (Xc, Ω), phase angle (phase angle, PhA, expressed in degrees), total body water (TBW, expressed in percentage), extracellular, water (ECW, expressed in percentage), fat mass (FM, expressed in percentage), FFM (expressed in percentage), and, finally, BCM (expressed in percentage). 

### 4.5. Ultrasonographic Evaluation

At the two study time points, T0 and T1 (after 12 weeks), all patients underwent an ultrasound examination of QRFT at 1/2 and 2/3. This exam represents an innovative diagnostic tool, useful to detect the loss of muscle mass in CKD patients. The ultrasonographic evaluation was carried out with the ultrasound equipment Esaote MyLab70 XVision (Genova, Italy) and the linear probe LA523, through B-mode modulation with a 7.5 MHz transducer. All evaluations were carried out by the same operator (A.N.). In order to perform the ultrasonographic evaluation of the QRFT at 1/2 and 2/3, three measurements were made bilaterally, in the supine position, with both knees in extension, at the level of two anatomical landmarks: the midpoint between the anterosuperior iliac spine and the upper limit of the patella (QRFT 1/2) and the boundary point between the lower third and the upper two-thirds of the quadriceps muscle (QRFT 2/3). The probe was placed perpendicular to the long axis of the muscle, covered with an abundant gel layer, on which minimal external pressure was exerted in order to prevent its compression [67,68]. 

### 4.6. Evaluation of Muscle Strength, Physical Performance and Flexibility

Functional assessments were carried out at baseline (T0) and after 12 weeks (T1).

(a) Muscle strength was evaluated through the hand grip ST, a dynamometer that evaluates the handgrip force (Jamar Plus, Performance Health (Warrenville, IL, USA). The seated patients were asked to squeeze the dynamometer as hard as possible with the elbow of the working hand at 90◦ close to the hip. The test was performed three times with both limbs, alternately, and the average value was considered. The cut-offs of hand grip ST are <30 kg for men and <20 kg for women [6].
(b) Physical performance was evaluated through three tests [69,70]:
  - The SPPB includes the gait speed (4 m walking), power (five-times chair sit to stand), and balance (tandem test). Each test is scored up to 4 points, and their sum indicates the level of performance. A sum of 12 points is the best score.
  - The SCPT analyses the power of the lower limbs. Patients have to climb 10 steps as fast as possible, without running or jumping. The time required to complete the task was recorded.
  - The SMWT evaluates the functional capacity. Patients have to walk, without running, for 6 min on 30 m of flat corridor. At the end of the test, the researchers recorded both the walking distance and the fatigue sensation through the BORG Scale (0–10) [71].
(c) For flexibility, the following tests were conducted:
  - The sit and reach test was used to evaluate the flexibility of the lower back and hamstring muscles. Patients were asked to sit on the floor with the feet placed against a box and to stretch, maintaining the legs straight. After two trials, the measurement of the third trial was recorded [72].
  - The back scratch test was used to assess upper limb mobility. The shoulder range of motion was measured by this test in standing position. The upper limb that is brought up performs a combined movement of flexion, extra-rotation ad abduction, while the other one that is brought down performs a combined movement of extension, extra-rotation and adduction. The distance between the 2 punches behind the back for both arms is recorded in centimeters [73].

### 4.7. Composition of the Functional Food Bars

As reported in the pilot study carried out by our research group, the two functional food bars used in this study were designed with innovative products obtained from circular agriculture [13]. The innovative ingredients previously chemically characterized were grape seed, grape pomace and olive leaf powders. These ingredients, together with the EVOO with a high content of minor polar compounds (MPCs) used for both formulations, were chosen for their high content of NBCs. Both bars were analyzed to evaluate the TAC (mgGAE/32g) using the Folin–Ciocalteu test and the percentage of antiradical activity (%AA) using the DPPH radical (2.2 -diphenyl-1-picrylhydrazyl). Bar 1 had a TAC of 209.54 mgGAE/32 g, and bar 2 had a TAC of 155.52 mgGAE/32 g. The %AA of bar 1 was 84.49%, while that for bar 2 was 80.53%.

### 4.8. Physical Exercise Protocol

The online training protocol combined resistance and aerobic exercises. It was performed three times per week, for 1 h in each session, using Microsoft Teams platform (24152.415.2975.367) for 12 weeks. The training program was supervised by a kinesiologist and was adapted every 4 weeks to increase volume and intensity of the session. Each session was structured as follows: (i) warm up of 10 min for mobility and balance; (ii) central session of 40 min of resistance and aerobic exercises; (iii) cool-down of 10 min. The RT phase involved both bodyweight and resistance band exercises. The selection of the appropriate elastic band for each patient was based on the study by Almeida Campos et al. 2020 [74]. The exercises were structured into two circuits of a maximum of 4 exercises, to be repeated 2–3 times. They involved the major muscle groups, with particular emphasis on the lower body and isolation exercises for deltoids, triceps, biceps and core. The intensity was increased every 4 weeks firstly through repetitions from 8 to 15, then increasing the time under tension and finally changing the resistance of the band. The AT involved movements with music at an intensity of 60–75% of heart rate reserve (HRR), calculated by the Karvonen formula, for each patient. During the first 2 weeks, the training was performed without music to allow patients to learn the choreography. Once they mastered the basic movements, music was introduced. The playlist for AT included tracks with a tempo of 120 beats per minute (bpm) to achieve an intensity of ≃60% of HRR that was maintained for 10 min in the first 2 weeks of training. Then, patients completed the training at ≃70–75% of HRR (128 bpm) for 20 min. Heart rate and Borg rating of the perceived exertion (RPE 0–10) were measured at the end of each circuit and aerobic exercise. The heart rate was monitored by personal smartwatches of patients (Polar M430). Each session ended with 5–10 min dedicated to stretching the main muscle groups involved during training.

### 4.9. Statistical Analysis

All data were entered into an Excel spreadsheet (Microsoft, Redmond, WA, USA), and the analysis was performed using the Windows Social Science Statistics Package, version 25.0 (IBM_SPSS, Chicago, IL, USA).

This pilot study was designed to collect information useful for defining the sample size of the definitive clinical trial. For this reason, we enrolled a total of 40 patients. 

The descriptive statistics analysis considered the mean ± standard deviation for the parameters with normal distribution (after confirmation with histograms and the Shapiro–Wilk test), while for the parameters with non-normal distribution, the analysis considered the median and minimum–maximum range. Subsequently, in the first case, the student *t*-test (parametric test) was applied, while in the second case, the Wilcoxon test (non-parametric test) was applied. A *p*-value < 0.05 was considered statistically significant for both tests. 

## 5. Conclusions

Our data support the thesis that the combination of FFs, containing NBCs, and a program of APA can counteract several comorbidities typical of CKD, such as arterial hypertension, dyslipidemia, US, etc. In fact, it has been stated that an exclusively pharmacological approach is not the optimal choice for the treatment of CKD-related complications. In order to strengthen the results obtained in this study, it would also be appropriate to implement additional blood chemistry parameters, such as venous bicarbonatemia, as well as monitoring additional biomarkers of inflammation, such as tumor necrosis factor (TNF)-α and IL-6, which could provide more detailed information about the systemic inflammatory state. 

Therefore, in clinical practice, it becomes fundamental to use a patient-holistic approach, involving not only nephrologists, but also clinical kinesiologists and nutritionists, trained for the management of nephropathic patients. Our study confirms the beneficial effects of NBCs present in secondary raw materials from circular agriculture. Future studies could be designed with other wastes and by-products rich in NBCs derived from other agri-food chains, in accordance with the European Green Deal to improve human health and QoL and, at the same time, protect the environment.

Finally, further studies, conducted on a wider population, would be necessary to support our findings.

## Figures and Tables

**Table 1:** Anthropometric and epidemiological features of the study populations.

Parameters		Mixed	APA	FFs	Controls
Age (years)	N	10	10	10	10
	T0	58.00 ± 8.25 ^a^	58.44 ± 8.28 ^a^	63.66 ± 4.61 ^a^	65.50 ± 4.43 ^a^
Gender (F/M)	N	10	10	10	10
	T0	2/8	3/7	5/5	3/7
Weight (kg)	N	10	10	10	10
	T0	84.72± 12.03 ^a^	75.98 ± 13.04 ^a^	75.20 ± 12.05 ^a^	71.73 ± 10.40 ^a^
BMI (kg/m^2^)	N	10	10	10	10
	T0	28.72 ± 4.02 ^a^	27.40 ± 4.29 ^a^	26.93 ± 3.48 ^a^	26.64 ± 3.62 ^a^

^a^ Data expressed as mean ± standard deviation. Abbreviations: APA, adapted physical activity group; BMI, body mass index; F, females; FFs, functional foods group; M, males.

**Table 2:** Laboratory parameters.

Laboratory Parameters		Mixed	APA	FFs	Controls
Hemoglobin (g/dL)	T0	14.05 ± 1.79 ^a^	14.14 ± 1.62 ^a^	13.42±1.66 ^a^	13.75 ± 1.86 ^a^
	T1	13.98 ± 1.79 ^a^	13.91 ± 1.55 ^a^	13.25 ± 1.70 ^a^	13.79 ± 1.78 ^a^
	*p*	n.s. ^b^	n.s. ^b^	n.s. ^b^	n.s. ^b^
Creatinine (mg/dL)	T0	1.41 ± 0.46 ^a^	1.50 ± 0.32 ^a^	1.72 ± 0.65 ^a^	1.51 ± 0.45 ^a^
	T1	1.57 ± 0.57 ^a^	1.66 ± 0.36 ^a^	1.80 ± 0.63 ^a^	1.57 ± 0.46 ^a^
	*p*	0.0144 ^b^	0.0009 ^b^	n.s. ^b^	n.s. ^b^
e-GFR (mL/min/1.73 m^2^) ^e^	T0	56.85 ± 20.97 ^a^	46.41 ± 10.83 ^a^	40.95 ± 15.03 ^a^	51.03 ± 18.29 ^a^
	T1	48.85 ± 15.22 ^a^	41.55 ± 10.32 ^a^	38.30 ± 13.44 ^a^	46.02 ± 12.98 ^a^
	*p*	n.s. ^b^	0.0003 ^b^	0.0350 ^b^	n.s. ^b^
Azotemia (mg/dL)	T0	59.11 ± 15.02 ^a^	47.44 ± 21.01 ^a^	57.00 ± 14.40 ^a^	61.22 ± 20.65 ^a^
	T1	54.00 ± 14.15 ^a^	50.78 ± 26.32 ^a^	52.00 ± 12.07 ^a^	67.32 ± 29.16 ^a^
	*p*	0.0272 ^b^	n.s. ^b^	n.s. ^b^	n.s. ^b^
Sodium (mEq/L)	T0	139.5 ± 3.75 ^a^	141.11 ± 2.09 ^a^	141.00 ± 1.41 ^a^	140.56 ± 2.19 ^a^
	T1	140.4 ± 1.71 ^a^	141.00 ± 1.66 ^a^	141.00 ± 1.50 ^a^	140.44 ± 3.78 ^a^
	*p*	n.s. ^b^	n.s. ^b^	n.s. ^b^	n.s. ^b^
Potassium (mEq/L)	T0	4.39 ± 0.32 ^a^	4.06 ± 0.43 ^a^	4.34 ± 0.36 ^a^	4.54 ± 0.28 ^a^
	T1	4.31 ± 0.46 ^a^	4.01 ± 0.52 ^a^	4.33 ± 0.35 ^a^	4.71 ± 0.62 ^a^
	*p*	n.s. ^b^	n.s. ^b^	n.s. ^b^	n.s. ^b^
Calcium (mg/dL)	T0	9.32 ± 0.33 ^a^	9.40 ± 0.50 ^a^	9.38 ± 0.43 ^a^	9.32 ± 0.44 ^a^
	T1	9.24 ± 0.33 ^a^	9.29 ± 0.63 ^a^	18.74 ± 27.48 ^a^	9.36 ± 0.59 ^a^
	*p*	n.s. ^b^	n.s. ^b^	n.s. ^b^	n.s. ^b^
Phosphorus (mg/dL)	T0	3.24 ± 0.46 ^a^	3.12 ± 0.58 ^a^	3.12 ± 0.44 ^a^	3.35 ± 0.71 ^a^
	T1	3.27 ± 0.29 ^a^	3.13 ± 0.60 ^a^	3.28 ± 0.56 ^a^	3.45 ± 0.71 ^a^
	*p*	n.s. ^b^	n.s. ^b^	n.s. ^b^	n.s. ^b^
TC (mg/dL)	T0	196 (170–240) ^c^	239 (85–333) ^c^	185 (126–229) ^c^	189.5 (136–221) ^c^
	T1	187.5 (153–240) ^c^	200 (69–282) ^c^	159 (133–196) ^c^	177.5 (115–222) ^c^
	*p*	n.s. ^d^	0.0039 ^d^	n.s. ^d^	n.s. ^d^
HDL-C (mg/dL)	T0	46 (35–73) ^c^	56.5 (37–75) ^c^	37 (27–67) ^c^	51 (35–63) ^c^
	T1	45 (32–66) ^c^	50.5 (31–73) ^c^	43 (27–67) ^c^	48 (34–64) ^c^
	*p*	n.s. ^d^	0.0156 ^d^	n.s. ^d^	n.s. ^d^
LDL-C (mg/dL)	T0	125.11 ± 29.87 ^a^	135.62 ± 60.36 ^a^	99.50 ± 26.11 ^a^	121.5 ± 28.82 ^a^
	T1	123.22 ± 21.69 ^a^	124.75 ± 57.76 ^a^	100.12 ± 17.68 ^a^	113.87 ± 36.92 ^a^
	*p*	n.s. ^b^	n.s. ^b^	n.s. ^b^	n.s. ^b^
Triglycerides (mg/dL)	T0	129.9 ± 73.01 ^a^	176.75 ± 121.85 ^a^	142.44 ± 59.19 ^a^	112.78 ± 35.89 ^a^
	T1	140.44 ± 101.43 ^a^	172.12 ± 160.58 ^a^	124.67 ± 53.02 ^a^	98.00 ± 35.46 ^a^
	*p*	n.s. ^b^	n.s. ^b^	n.s. ^b^	n.s. ^b^
Albuminemia (gr/dL)	T0	4.74 ± 0.11 ^a^	4.61 ± 0.34 ^a^	4.70 ± 0.31 ^a^	4.58 ± 0.23 ^a^
	T1	4.59 ± 0.20 ^a^	4.56 ± 0.40 ^a^	4.65 ± 0.39 ^a^	4.67 ± 0.40 ^a^
	*p*	n.s. ^b^	n.s. ^b^	n.s. ^b^	n.s. ^b^
Albuminuria (mg/gr) ^f^	T0	100.45 ± 119.95 ^a^	206.06 ± 412.21 ^a^	171.16 ± 253.04 ^a^	80.60 ± 66.23 ^a^
	T1	71.05 ± 103.97 ^a^	103.07 ± 174.35 ^a^	156.62 ± 235.89 ^a^	67.86 ± 51.39 ^a^
	*p*	n.s. ^b^	n.s. ^b^	n.s. ^b^	n.s. ^b^
Uricemia (mg/dL)	T0	6.30 ± 1.15 ^a^	6.59 ± 1.10 ^a^	5.82 ± 0.84 ^a^	5.97 ± 1.40 ^a^
	T1	6.36 ± 1.07 ^a^	6.64 ± 2.14 ^a^	6.05 ± 0.89 ^a^	5.64 ± 1.79 ^a^
	*p*	n.s. ^b^	n.s. ^b^	n.s. ^b^	n.s. ^b^

^a^ Data expressed as mean ± standard deviation. ^b^ Applied test: *t*-test for paired data; ^c^ Data expressed as median, and the minimum–maximum range is shown in brackets. ^d^ Applied test: Wilcoxon test. ^e^ eGFR was calculated by chronic kidney disease-epidemiology collaboration (CKD-EPI) formula. ^f^ Albuminuria was calculated by albumin-creatinine ratio (ACR) in a morning urine sample (spot). Values of *p* ≤ 0.05 are considered statistically significant. Abbreviations: APA, adapted physical activity group; e-GFR, estimated glomerular filtration rate; FFs, functional foods group; HDL-C, high-density lipoprotein cholesterol; LDL-C, low-density lipoprotein cholesterol; TC, total cholesterol, n.s., not significant.

**Table 3:** Arterial blood pressure parameters.

Arterial Blood Pressure Parameters		Mixed	APA	FFs	Controls
Systolic Pressure (mmHg)	T0	135.60 ± 22.32 ^a^	132.89 ± 13.18 ^a^	140.89 ± 12.78 ^a^	137.80 ± 17.13 ^a^
	T1	129.30 ± 14.97 ^a^	129.22 ± 18.80 ^a^	136.78 ± 10.71 ^a^	139.40 ± 16.17 ^a^
	*p*	n.s. ^b^	n.s. ^b^	n.s. ^b^	n.s. ^b^
Diastolic Pressure (mmHg)	T0	88.50 ± 15.28 ^a^	82.00 ± 12.84 ^a^	84.78 ± 9.35 ^a^	78.30 ± 9.57 ^a^
	T1	78.00 ± 9.77 ^a^	75.78 ± 9.36 ^a^	80.56 ± 5.17 ^a^	78.50 ± 8.83 ^a^
	*p*	0.0169 ^b^	n.s. ^b^	n.s. ^b^	n.s. ^b^

^a^ Data expressed as mean ± standard deviation; ^b^ Applied test: *t*-test for paired data. Values of *p* ≤ 0.05 are considered statistically significant. Abbreviations: APA, adapted physical activity group; FFs, functional foods group; n.s., not significant.

**Table 4:** Inflammatory and oxidative stress parameters.

Biomarkers of Inflammation and Oxidative Stress		Mixed	APA	FFs	Controls
FORT (U)	T0	271.40 ± 147.93 ^a^	311.78 ± 150.41 ^a^	287.22 ± 112.15 ^a^	347.33 ± 140.07 ^a^
	T1	336.70 ± 202.38 ^a^	312.33 ± 185.63 ^a^	283.00 ± 182.67 ^a^	254.33 ± 145.11 ^a^
	*p*	n.s. ^b^	n.s. ^b^	n.s. ^b^	n.s. ^b^
FORD (mmol/L Trolox equivalents)	T0	1.41 ± 0.47 ^a^	1.38 ± 0.68 ^a^	1.19 ± 0.29 ^a^	1.18 ± 0.56 ^a^
	T1	1.89 ± 0.54 ^a^	1.22 ± 0.55 ^a^	0.86 ± 0.48 ^a^	1.26 ± 0.35 ^a^
	*p*	0.0203 ^b^	n.s. ^b^	n.s. ^b^	n.s. ^b^
CRP (mg/L)	T0	3.8 (0.4–9.5) ^c^	1.55 (1–5.1) ^c^	2.5 (0.4–7.9) ^c^	1.1 (0.5–5) ^c^
	T1	1.9 (0.4–5) ^c^	1.65 (0.8–5.6) ^c^	1.7 (0.6–8) ^c^	1.0 (0.5–7.5) ^c^
	*p*	0.0313 ^d^	n.s. ^d^	n.s. ^d^	n.s. ^d^
ESR (mm/h)	T0	26.67 ± 16.72 ^a^	38.00 ± 18.48 ^a^	29.20 ± 33.57 ^a^	33.89 ± 25.14 ^a^
	T1	27.22 ± 15.31 ^a^	37.33 ± 18.12 ^a^	26.80 ± 27.64 ^a^	34.55 ± 23.20 ^a^
	*p*	n.s. ^b^	n.s. ^b^	n.s. ^b^	n.s. ^b^

^a^ Data expressed as mean ± standard deviation; ^b^ Applied test: *t*-test for paired data; ^c^ Data expressed as median, and the minimum–maximum range is shown in brackets; ^d^ Applied test: Wilcoxon test. Values of *p* ≤ 0.05 are considered statistically significant. Abbreviations: APA, adapted physical activity group; ESR, erythrocyte sedimentation rate; FFs, functional foods group; FORD, free oxygen radicals defense; FORT, free oxygen radicals test; CRP, C-reactive protein; n.s., not significant.

**Table 5:** Body composition and ultrasonographic parameters.

Anthropometric Body Composition and Ultrasonographic Evaluation Parameters		Mixed	APA	FFs	Controls
Weight (kg)	T0	84.72 ± 12.03 ^a^	75.98 ± 13.04 ^a^	75.20 ± 12.05 ^a^	71.73 ± 10.40 ^a^
	T1	84.08 ± 11.85 ^a^	75.37 ± 12.98 ^a^	75.49 ± 11.51 ^a^	72.20 ± 10.52 ^a^
	*p*	n.s. ^b^	n.s. ^b^	n.s. ^b^	n.s. ^b^
BMI (kg/m^2^)	T0	28.72 ± 4.02 ^a^	27.40 ± 4.29 ^a^	26.93 ± 3.48 ^a^	26.64 ± 3.62 ^a^
	T1	28.68 ± 4.14 ^a^	27.23 ± 4.54 ^a^	27.04 ± 3.39 ^a^	26.84 ± 3.84 ^a^
	*p*	n.s. ^b^	n.s. ^b^	n.s. ^b^	n.s. ^b^
Resistance (Ω)	T0	464.5 ± 60.29 ^a^	518.78 ± 74.71 ^a^	517.62 ± 62.66 ^a^	490.90 ± 107.24 ^a^
	T1	466.40 ± 60.07 ^a^	479.00 ± 47.22 ^a^	517.87 ± 92.06 ^a^	490.90 ± 96.41 ^a^
	*p*	n.s. ^b^	0.0491 ^b^	n.s. ^b^	n.s. ^b^
Reactance (Ω)	T0	39.80 ± 5.67 ^a^	49.89 ± 8.15 ^a^	45.22 ± 6.73 ^a^	41.5 ± 6.45 ^a^
	T1	45.40 ± 8.03 ^a^	52.00 ± 9.38 ^a^	47.75 ± 10.46 ^a^	44.9 ± 6.19 ^a^
	*p*	0.0232 ^b^	n.s. ^b^	n.s. ^b^	n.s. ^b^
Phase angle (°)	T0	5.56 ± 0.81 ^a^	5.73 ± 0.85 ^a^	4.92 ± 0.73 ^a^	4.99 ± 1.00 ^a^
	T1	5.06 ± 0.89 ^a^	5.63 ± 0.40 ^a^	5.60 ± 1.08 ^a^	5.33 ± 0.94 ^a^
	*p*	n.s. ^b^	n.s. ^b^	n.s. ^b^	n.s. ^b^
TBW (%)	T0	53.89 ± 4.82 ^a^	52.60 ± 5.43 ^a^	53.31 ± 5.96 ^a^	56.47 ± 7.86 ^a^
	T1	53.99 ± 4.65 ^a^	54.20 ± 5.61 ^a^	54.71 ± 7.50 ^a^	56.17 ± 6.42 ^a^
	*p*	n.s. ^b^	n.s. ^b^	n.s. ^b^	n.s. ^b^
ECW (%)	T0	50.86 ± 4.44 ^a^	47.14 ± 3.61 ^a^	50.94 ± 4.35 ^a^	51.59 ± 6.73 ^a^
	T1	47.87 ± 4.68 ^a^	45.81 ± 2.10 ^a^	49.51 ± 3.86 ^a^	49.39 ± 5.21 ^a^
	*p*	n.s. ^b^	n.s. ^b^	n.s. ^b^	n.s. ^b^
FM (%)	T0	27.00 ± 6.32 ^a^	29.01 ± 7.37 ^a^	27.84 ± 8.50 ^a^	24.22 ± 11.68 ^a^
	T1	25.92 ± 5.66 ^a^	25.77 ± 6.55 ^a^	27.36 ± 10.03 ^a^	28.02 ± 17.09 ^a^
	*p*	n.s. ^b^	n.s. ^b^	n.s. ^b^	n.s. ^b^
FFM (%)	T0	69.36 ± 7.27 ^a^	72.02 ± 7.08 ^a^	72.16 ± 8.50 ^a^	75.78 ± 11.68 ^a^
	T1	74.14 ± 5.55 ^a^	73.41 ± 7.14 ^a^	72.63 ± 10.03 ^a^	75.94 ± 9.02 ^a^
	*p*	0.0258 ^b^	n.s. ^b^	n.s. ^b^	n.s. ^b^
BCM (%)	T0	48.51 ± 4.48 ^a^	51.34 ± 1.91 ^a^	48.17 ± 4.59 ^a^	47.48 ± 7.11 ^a^
	T1	50.90 ± 5.26 ^a^	54.27 ± 3.69 ^a^	49.70 ± 4.10 ^a^	49.82 ± 5.52 ^a^
	*p*	n.s. ^b^	0.0270 ^b^	n.s. ^b^	n.s. ^b^
QRFT left 1/2 (cm)	T0	1.36 ± 0.38 ^a^	1.58 ± 0.15 ^a^	1.47 ± 0.47 ^a^	1.64 ± 0.34 ^a^
	T1	1.47 ± 0.61 ^a^	1.77 ± 0.25 ^a^	1.48 ± 0.49 ^a^	1.54 ± 0.40 ^a^
	*p*	n.s. ^b^	0.0465 ^b^	n.s. ^b^	n.s. ^b^
QRFT right 1/2 (cm)	T0	1.33 ± 0.40 ^a^	1.46 ± 0.32 ^a^	1.33 ± 0.38 ^a^	1.47 ± 0.31 ^a^
	T1	1.49 ± 0.64 ^a^	1.76 ± 0.48 ^a^	1.38 ± 0.39 ^a^	1.54 ± 0.31 ^a^
	*p*	n.s. ^b^	0.0255 ^b^	n.s. ^b^	n.s. ^b^
QRFT left 2/3 (cm)	T0	1.21 ± 0.58 ^a^	1.59 ± 0.30 ^a^	1.27 ± 0.43 ^a^	1.51 ± 0.40 ^a^
	T1	1.33 ± 0.54 ^a^	1.79 ± 0.34 ^a^	1.25 ± 0.43 ^a^	1.43 ± 0.35 ^a^
	*p*	n.s. ^b^	0.0480 ^b^	n.s. ^b^	n.s. ^b^
QRFT right 2/3 (cm)	T0	1.23 ± 0.59 ^a^	1.48 ± 0.38 ^a^	1.24 ± 0.49 ^a^	1.57 ± 0.44 ^a^
	T1	1.37 ± 0.55 ^a^	1.66 ± 0.45 ^a^	1.32 ± 0.41 ^a^	1.41 ± 0.43 ^a^
	*p*	n.s. ^b^	n.s. ^b^	n.s. ^b^	0.0435 ^b^

^a^ Data expressed as mean ± standard deviation; ^b^ Applied test: *t*-test for paired data. Values of *p* ≤ 0.05 are considered statistically significant. Abbreviations: APA, adapted physical activity group; BMI, body mass index; TBW, total body water; ECW, extracellular water; FFs, functional foods group; FM, fat mass; FFM, fat free mass; BCM, body cell mass; QRFT, quadriceps rectus femoris thickness; n.s., not significant.

**Table 6:** Physical performance and muscle strength parameters.

Evaluation of Physical Performance and Muscle Strength		Mixed	APA	FFs	Controls
SPPB (points)	T0	11.70 ± 0.95 ^a^	11.56 ± 0.73 ^a^	9.89 ± 1.83 ^a^	10.80 ± 1.40 ^a^
	T1	12.00 ± 0.00 ^a^	11.89 ± 0.33 ^a^	11.33 ± 0.87 ^a^	10.50 ± 2.07 ^a^
	*p*	n.s. ^b^	n.s. ^b^	0.0316 ^b^	n.s. ^b^
SMWT (m)	T0	572.00 ± 75.98 ^a^	603.75 ± 107.03 ^a^	473.61 ± 152.27 ^a^	504.00 ± 145.37 ^a^
	T1	624.50 ± 89.15 ^a^	636.87 ± 97.13 ^a^	502.67 ± 108.16 ^a^	505.50 ± 151.30 ^a^
	*p*	0.0002 ^b^	0.0070 ^b^	n.s. ^b^	n.s. ^b^
SMWT BORG (points)	T0	2.50 ± 1.33 ^a^	3.22 ± 1.20 ^a^	2.56 ± 1.01 ^a^	2.90 ± 1.20 ^a^
	T1	3.10 ± 1.33 ^a^	2.94 ± 1.01 ^a^	2.50 ± 0.87 ^a^	2.70 ± 1.51 ^a^
	*p*	n.s. ^b^	n.s. ^b^	n.s. ^b^	n.s. ^b^
SCPT (W)	T0	357.20 ± 113.04 ^a^	371. 21 ± 128.80 ^a^	192.70 ± 97.57 ^a^	258.40 ± 81.59 ^a^
	T1	363.04 ± 77.61 ^a^	345.43 ± 85.75 ^a^	227.53 ± 109.02 ^a^	270.80 ± 69.95 ^a^
	*p*	n.s. ^b^	n.s. ^b^	0.0280 ^b^	n.s. ^b^
HGST right (kg)	T0	41.84 ± 13.14 ^a^	40.01 ± 12.48 ^a^	31.98 ± 13.99 ^a^	32.50 ± 10.21 ^a^
	T1	43.53 ± 14.98 ^a^	43.03 ± 14.70 ^a^	34.47 ± 11.96 ^a^	31.60 ± 11.35 ^a^
	*p*	n.s. ^b^	n.s. ^b^	0.0500 ^b^	n.s. ^b^
HGST left (kg)	T0	38.83 ± 13.68 ^a^	36.85 ± 11.18 ^a^	28.84 ± 11.05 ^a^	29.65 ± 10.70 ^a^
	T1	41.25 ± 13.79 ^a^	39.30 ± 12.47 ^a^	29.43 ± 10.09 ^a^	30.30 ± 12.30 ^a^
	*p*	0.0491 ^b^	n.s. ^b^	n.s. ^b^	n.s. ^b^
Scratch test right (cm)	T0	29.90 ± 9.05 ^a^	23.56 ± 10.27 ^a^	39.89 ± 10.86 ^a^	38.10 ± 10.64 ^a^
	T1	30.40 ± 6.20 ^a^	22.72 ± 9.50 ^a^	33.89 ± 8.12 ^a^	34.00 ± 6.25 ^a^
	*p*	n.s. ^b^	n.s. ^b^	n.s. ^b^	n.s. ^b^
Scratch test left (cm)	T0	27.50 ± 6.19 ^a^	28.00 ± 8.65 ^a^	43.28 ± 9.69 ^a^	41.80 ± 11.64 ^a^
	T1	27.90 ± 6.85 ^a^	27.44 ± 8.14 ^a^	36.78 ± 9.54 ^a^	37.90 ± 6.37 ^a^
	*p*	n.s. ^b^	n.s. ^b^	n.s. ^b^	n.s. ^b^
Sit and reach (cm)	T0	−6.15 ± 12.05 ^a^	−6.56 ± 6.82 ^a^	−14.00 ± 11.48 ^a^	−10.90 ± 8.16 ^a^
	T1	−2.75 ± 5.98 ^a^	−2.67 ± 4.76 ^a^	−11.37 ± 13.00 ^a^	−10.60 ± 8.14 ^a^
	*p*	n.s. ^b^	n.s. ^b^	n.s. ^b^	n.s. ^b^

^a^ Data expressed as mean ± standard deviation; ^b^ Applied test: *t*-test for paired data. Values of *p* ≤ 0.05 are considered statistically significant. Abbreviations: APA, adapted physical activity group; FFs, functional foods group; HGST, handgrip strength test; SCPT, stair climb power test; SMWT, six-minute walking test; SPPB, short physical performance battery; n.s., not significant.

## Data Availability

The original contributions presented in the study are included in the article, further inquiries can be directed to the corresponding author.
